# Serum and ascites tumor markers in the diagnostic and prognostic prediction for appendiceal pseudomyxoma peritonei

**DOI:** 10.1186/s12885-023-10545-7

**Published:** 2023-01-26

**Authors:** Bing Wang, Ruiqing Ma, Benqiang Rao, Hongbin Xu

**Affiliations:** 1 Department of Myxoma, Aerospace Center Hospital, 15 Yuquan Road, Haidian 100049 Beijing, China; 2 Department of Gastrointestinal Surgery, Beijing Shijitan Hospital, Capital Medical University, 10 Tieyi Road, Haidian 100038 Beijing, China

**Keywords:** Appendiceal pseudomyxoma peritonei, Ascites tumor biomarkers, Serum tumor biomarkers, Diagnosis, Prognosis

## Abstract

**Background:**

To investigate the expression of carcinoembryonic antigen (CEA), cancer antigen 199 (CA199) and CA125 in serum and ascites of appendiceal pseudomyxoma peritonei (PMP) patients relative to their diagnostic and predictive value.

**Methods:**

The study comprised 183 patients with pathologically confirmed appendiceal PMP, enrolled from May 2012 to June 2020, in Aerospace Center Hospital. Serum and ascites tumor markers were obtained, and their diagnostic values were compared by receiver operating characteristic (ROC) curves. The prognostic factors of appendiceal PMP with different pathologic subgroups were calculated by univariate and multivariate Cox proportional hazard regression models.

**Results:**

There were significant differences between the numbers of patients with positive CEA and CA199 in serum vs. ascites: *p* = 0.034 in CEA and *p* = 0.006 in CA199, respectively. The sensitivities with optimal cut-off values for ascites markers of CEA, CA199 and CA125 were 83.5%, 88.9% and 72.6%, respectively. CEA in ascites showed significant difference in the diagnosis of appendiceal PMP (*p* = 0.000); the areas under the ROC curves (AUROCs) and specificity were 0.725, 70.7%, respectively. Univariate analysis showed that the higher the ascites tumor markers, the poorer the survival (*p* = 0.014). Multivariate analysis indicated that completeness of cytoreduction (CCR), ascites CEA and pathological grade were independent risk factors for overall survival (OS).

**Conclusion:**

CEA in ascites can be used to help specify the origin of PMP. Furthermore, elevation of ascites CEA, high pathological grade and incomplete cytoreduction predicted poor prognosis of appendiceal PMP.

## Introduction

Pseudomyxoma peritonei (PMP), often called “jelly-belly”, is a rare peritoneal malignancy that develops from a perforated epithelial tumor of the appendix, characterized by diffuse, progressive mucinous ascites. Clinically, it may be discovered incidentally by abdominal imaging or surgically. However, symptoms of increased abdominal girth, intestinal obstruction and malnutrition can call attention to the accumulation of large amounts of mucinous tumor [1–3]. When the appendix ruptures, tumor cells spread throughout the abdominal cavity via paths of fluid re-absorption [4]. Sugarbaker’s cytoreductive surgery (CRS) combined with hyperthermic intraperitoneal chemotherapy (HIPEC) is considered the current standard of treatment [5–7]. According to the 2016 Peritoneal Surface Oncology Group International (PSOGI) criteria, pathological diagnosis for PMP was classified into four categories: acellular mucin (AC), low-grade mucinous carcinoma peritonei (LG-MCP), high-grade mucinous carcinoma peritonei (HG-MCP), and high-grade mucinous carcinoma peritonei with signet ring cells (HGMC-S) [2].

The application of the serum markers carcinoembryonic antigen (CEA), cancer antigen 199 (CA19-9) and CA125 in colorectal, pancreatic, and ovarian cancer has been widely reported in the literature [8–10]. Moreover, other studies have reported the application of serum markers in the diagnosis and prognosis of appendiceal PMP [11–13]. However, there are few relevant reports about ascites markers in appendiceal PMP.

In the present study, following laparotomy, ascites was collected for tumor marker testing. If the ascites was a viscous liquid, it required filtration. For the patients without ascites, saline was used to lavage the abdominopelvic cavity, then aspirated and sent for tumor marker examination. A previous study reported that the immune response of markers such as CEA also existed in mucus, suggesting that this kind of cell membrane glycoprotein could fall off the surface of the PMP cell membrane into mucinous ascites [14]. Therefore, it could be speculated that ascites tumor markers would more likely be positive. This might have an important role in the early diagnosis of PMP and potentially improve the prognosis.

The aim of the current study was to survey the expression of CEA, CA199 and CA125 in both serum and ascites to determine their value in the diagnosis and prediction of prognosis of appendiceal PMP.

## Patients and methods

### Patients

The medical records from a database of patients with PMP who attended the Aerospace Center Hospital, Beijing, China between May 2012 and June 2020 were retrospectively reviewed. Inclusion criteria were: (1) diagnosis of appendiceal PMP on histology and histopathologic subtype confirmed by two experienced pathologists; and (2) treatment with cytoreductive surgery (CRS) and hyperthermic intraperitoneal chemotherapy (HIPEC). Exclusion criteria were: (1) lost to follow-up; (2) incomplete medical records; or (3) history of other severe organic disease. A total of 183 patients were included in the final analysis.

In order to test the specificity of ascites tumor markers in the diagnosis of appendiceal PMP, our study included 98 patients with peritoneal surface malignancies originating from extra-appendix as a control group. There were 34 cases of peritoneal malignant mesothelioma, 28 cases of colorectal mucinous adenocarcinoma, 19 cases of ovarian mucinous carcinoma, 17 cases of ovarian serous carcinoma. All patients treated with CRS and HIPEC. The medical records were complete.

### Surgical treatment

Following laparotomy, ascites was obtained and sent for tumor biomarker testing, and an intraoperative peritoneal cancer index (PCI) was determined according to the evaluation criteria described by Professor Sugarbaker [15]. This index first divided the abdomen, pelvic cavity and small intestine into 13 regions, each of which was scored from 0 to 3 points according to the maximum diameter of the tumor. The highest total score was 39 points.

After PCI evaluation, we employed professor Sugarbaker’s CRS procedure [16] to remove tumor tissues visible to the naked eye as much as possible throughout the peritoneum, combined as necessary with organ resection. Following completion of the surgical CRS, the residual tumor load was evaluated (postoperative PCI), and a completeness of cytoreduction (CCR) score was calculated. CCR was again scored on a scale from 0 to 3: CCR-0, no macroscopic residual cancer remained; CCR-1, residual tumor nodules < 2.5 mm; CCR-2, nodules between 2.5 mm and 2.5 cm remained; and CCR-3, tumor nodules > 2.5 cm remained [15].

HIPEC was conducted in all patients for 60 min using a closed-abdomen technique with mitomycin (20 mg/m^2^). An extracorporeal device maintained intraabdominal temperature between 41 and 42 °C.

### Study parameters

The analysis included the following clinicopathological parameters: gender, age, overall survival (OS), survival rate, intraoperative PCI, CCR, serum CEA, serum CA199, serum CA125, ascites CEA, ascites CA199, ascites CA125, pathological grade, and follow-up time. The serum CEA cut-off value was set at 10 ng/ml, CA125 at 35 U/ml, and CA199 at 37 U/ml.

### Follow-up

The patients were reexamined every 6 months, including abdominopelvic enhanced CT and serum tumor markers. Follow-up methods included telephone and reexamination. The follow-up time extended from the initial operative date to June 2020, and the OS was determined. Median follow-up after CRS was 41 months (range, 5 to 235 months). Follow-up was obtained on all patients.

### Statistical analysis

Categorical variables were described using frequency (percentage). Continuous variables were described using median [interquartile range (IQR)]. The receiver operating characteristic (ROC) curves was used to show the sensitivity and specificity in the diagnosis of PMP between serum and ascites markers. The optimal cut-off values were calculated with the criteria of Youden’s index. Univariate survival analysis was performed with the Kaplan-Meier method and the log-rank test. Statistically significant variables were included in a multivariate analysis, which used a Cox proportional hazards model to identify independent prognostic factors for survival. Because all patients diagnosed as AC survived postoperatively, the data in this group did not show statistical significance in the univariate survival analysis. To test the specificity of ascites tumor markers, these markers were determined in patients with peritoneal surface malignancies whose diagnosis were extra-appendix origin as a control group. All live patients were censored. *P* < 0.05 was considered statistically significant. The data analyses were performed using SPSS 20.0 (SPSS, Chicago, Illinois, USA).

## Results

### Clinicopathological characteristics

Demographic and clinical characteristics of the 183 patients are presented in Table 1. There were 70 (38%) males and 113 (62%) females, with a median age of 56 years (range, 19 to 77 years). Serum CEA, CA199, and CA125 were elevated in 122 (67%), 107 (58%), and 113 (62%) patients, respectively. Ascites CEA, CA199, and CA125 were elevated in 151 (83%), 132 (72%), and 143 (78%) patients, respectively. Intraoperative PCI scores were as follows: <20 in 62 (34%) patients, and ≥ 20 score in 121 (66%) patients. CCR scores were as follows: 0/1 in 95 (52%) patients, and 2/3 in 88 (48%) patients. Pathological diagnosis showed 13 (7%) patients had AC, 106 (58%) patients had LG-MCP, 36 (20%) patients had HG-MCP, and 28 (15%) patients had HGMC-S, respectively.

**Table 1:** Patients’ clinical and demographic data (*n* = 183)

Characteristics	No. of patients
Gender	
Male	70 (38%)
Female	113 (62%)
Age at hospitalization (years)	
Median (range)	56 (19-77)
<56	52 (28%)
≥56	131 (72%)
Elevated serum tumor markers	
CEA	122 (67%)
CA199	107 (58%)
CA125	113 (62%)
Intraoperative PCI	
Median (range)	25 (0-39)
<20	62 (34%)
≥20	121 (66%)
CCR	
Median (range)	2 (0-3)
0/1	95 (52%)
2/3	88 (48%)
Elevated ascites tumor markers	
CEA	151 (83%)
CA199	132 (72%)
CA125	143 (78%)
Any one elevated	10 (5%)
Any two elevated	36 (20%)
All three elevated	115 (63%)
Pathological grade	
AC	13 (7%)
LG-MCP	106 (58%)
HG-MCP	36 (20%)
HGMC-S	28 (15%)

*PCI* peritoneal cancer index, *CCR* completeness of cytoreduction, *AC* acellular mucin, *LG-MCP* low-grade mucinous carcinoma peritonei, *HG-MCP* high-grade mucinous carcinoma peritonei, *HGMC-S* high-grade mucinous carcinoma peritonei with signet ring cells

### The sensitivity of serum and ascites tumor markers in the diagnosis of appendiceal PMP

**Fig. 1:**
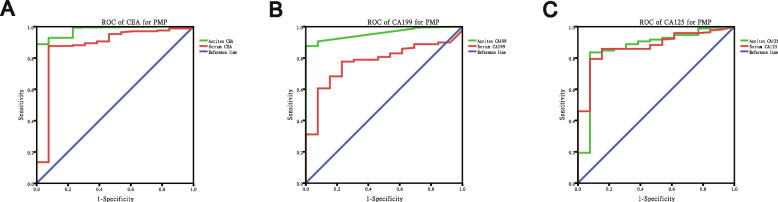
Sensitivity comparison of ascites tumor markers and serum tumor markers in the diagnosis of appendiceal PMP (**A**) CEA, *p* = 0.034 (**B**) CA199, *p* = 0.006 (**C**) CA125, *p* = 0.683

As shown in Fig. 1; Table 2, the areas under the ROC curves (AUROCs) in the diagnosis of appendiceal PMP using CEA were 0.876 and 0.979 in serum and ascites markers, respectively. Similarly, the diagnostic AUROCs using CA199 were 0.774 and 0.957, and the diagnostic AUROCs using CA125 were 0.872 and 0.866 in serum and ascites markers, respectively. There were significant differences in the diagnosis of appendiceal PMP between serum markers and ascites markers (CEA, *p* = 0.034; CA199, *p* = 0.006), respectively. But CA125 was not significant different (CA125, *p* = 0.683). The optimal cut-off values for ascites markers of CEA, CA199 and CA125 were 97 ng/ml, 145 U/ml and 173 U/ml, respectively. The sensitivities with optimal cut-off values of CEA, CA199 and CA125 were 83.5%, 88.9% and 72.6% in ascites markers, respectively. The specificities of CEA, CA199 and CA125 were 70.7%, 46.8% and 42.5% in ascites markers, respectively.

**Table 2:** General data of ascites tumor markers

Tumor markers	AUC-ROC	Optimal cut-off	Sensitivity	Specificity
Ascites CEA	0.979	97 ng / ml	83.5%	70.7%
Ascites CA199	0.957	145 U / ml	88.9%	46.8%
Ascites CA125	0.866	173 U / ml	72.6%	42.5%

*AUC-ROC* area under the receiver operating characteristics curve

### The specificity of ascites tumor markers in the diagnosis of appendiceal PMP

**Fig. 2:**
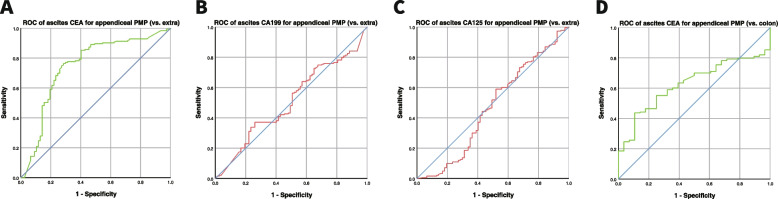
Specificity of ascites tumor markers in the diagnosis of appendiceal PMP (**A**) CEA, *p* = 0.000 (**B**) CA199, *p* = 0.602 (**C**) CA125, *p* = 0.180 (**D**) CEA, *p* = 0.030

To test specificity, ascites tumor markers of pathologically proven extra-appendix origin were used as a control group, comprised of 98 patients with a median age of 56 years. The AUROCs in the diagnosis of appendiceal PMP using CEA, CA199 and CA125 were 0.747, 0.509 and 0.469 in ascites markers, respectively. Ascites CEA showed significant difference in the diagnosis of appendiceal PMP (*p* = 0.000). There was no significant difference in the other two ascites tumor markers (CA199, *p* = 0.816; CA125, *p* = 0.426), respectively. The specificities were 79.2%, 49.4% and 50.6%, respectively. To further verify whether CEA of ascites can help diagnose the origin of appendiceal PMP, we selected colonic mucinous adenocarcinoma, which is closest to the appendix, as a control. The results showed that the AUROC in the diagnosis of appendiceal PMP using ascites CEA was 0.627 and were statistically different (*P* = 0.030). The sensitivity and specificity were 53.6% and 75.0%, respectively (Fig. 2).

### Univariate and multivariate logistic regression of clinicopathological characteristics for appendiceal PMP

Univariate analysis suggested that CCR, serum CA199, serum CA125, ascites CEA, ascites CA125 and pathological grade were the predictors of OS for appendiceal PMP (Table 3). Multivariate analysis of the statistically significant factors by univariate analysis indicated that CCR, ascites CEA and pathological grade were independent risk factors for OS (Table 4).

**Table 3:** Univariate analysis of OS after CRS (*n* = 183)

Variables	Log-Rank *P* value of univariate analysis				
	Overall	AC	LG-MCP	HG-MCP	HGMC-S
	*P* value	*P* value	*P* value	*P* value	*P* value
Gender (Male vs. Female)	0.210	-	0.635	0.022*	0.469
Age (<56 vs. ≥56, years)	0.512	-	0.672	0.566	0.349
Intraoperative PCI (<25 vs. ≥25)	0.781	-	0.975	0.630	0.668
CCR (0/1 vs. 2/3)	0.025*	-	0.217	0.711	0.086
Serum CEA (<10 vs. ≥10, ng/ml)	0.220	-	0.988	0.725	0.033*
Serum CA199 (<37 vs. ≥37, U/ml)	0.015*	-	0.417	0.277	0.802
Serum CA125 (<35 vs. ≥35, U/ml)	0.002*	-	0.069	0.262	0.553
Ascites CEA (<97 vs. ≥97, ng/ml)	0.024*	-	0.671	0.486	0.153
Ascites CA199 (<145 vs. ≥145, U/ml)	0.075	-	0.572	0.587	0.250
Ascites CA125 (<173 vs. ≥173, U/ml)	0.023*	-	0.511	0.349	0.496
Pathological grade (AC vs. LG-MCP vs. HG-MCP vs. HGMC-S)	0.000*				

*OS* overall survival, *CRS* cytoreductive surgery, *PCI* peritoneal cancer index, *CCR* completeness of cytoreduction, *AC* acellular mucin, *LG-MCP* low-grade mucinous carcinoma peritonei, *HG-MCP* high-grade mucinous carcinoma peritonei, *HGMC-S* high-grade mucinous carcinoma peritonei with signet ring cells

^*^P <0.05

**Table 4:** Multivariate analysis for statistically significant results (*n* = 183)

Variables	B	SE	Wald	*P* value	Exp(B)	95.0% CI for Exp(B)	
						Bottom	Upper
CCR	.608	.162	14.048	.000	1.836	1.336	2.523
Serum CA199	.000	.000	.870	.351	1.000	1.000	1.000
Serum CA125	.000	.001	.076	.783	1.000	.999	1.001
Ascites CEA	.703	.158	19.696	.000	2.054	1.479	2.692
Ascites CA125	.000	.000	.043	.836	1.000	1.000	1.000
Pathological grade	.769	.136	31.824	.000	2.158	1.652	2.819

*CCR* completeness of cytoreduction, *B* unstandardized coefficient, *SE* standard error, *Wald* Wald 's test, *Exp* odds ratio, *CI* confidence intervals

### The overall survival in appendiceal PMP patients of elevated markers in serum and ascites

**Fig. 3:**
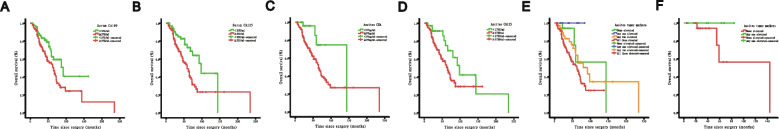
Overall survival curves of appendiceal PMP patients in different tumor markers (**A**) serum CA199, *p* = 0.015 (**B**) serum CA125, *p* = 0.002 (**C**) ascites CEA, *p* = 0.024 (**D**) ascites CA125, *p* = 0.023 (**E**) with elevated ascites tumor markers or without, *p* = 0.014 (**F**) comparison of none elevated ascites markers and any one elevated markers, *p* = 0.104

Patients with elevated serum markers CA199 and CA125 had poor survival (CA199, *p* = 0.015; CA125, *p* = 0.002; Fig. 3). Additionally, patients with elevated ascites markers CEA and CA125 had poor survival (CEA, *p* = 0.024; CA125, *p* = 0.023). The study population was divided into four groups according to the number of elevated ascites tumor markers (from 0 to 3). The univariate analysis showed that the more numbers of elevated ascites tumor markers the poorer survival (*p* = 0.014). We also analyzed the prognostic factors of none elevated ascites markers and any one elevated biomarker. The result showed no statistical significance (*p* = 0.104).

## Discussion

### Analysis of diagnosis and prognostic factors in patients with PMP

The diagnosis of PMP principally includes imaging examination and serum tumor markers. However, with negative serum tumor markers in most patients and the precise origin of PMP that can remain in doubt, especially in female patients, clinicians can be left in a quandary. In this study, the ascites tumor markers proved more sensitive than serum tumor markers. Ascites CEA could assist in defining the origin of PMP. The difference in CEA of ascites in the diagnosis of appendiceal PMP and colonic mucinous adenocarcinoma may be related to the clinical features of PMP leading to a large collection of mucinous ascites. However, to further diagnose appendiceal PMP, we also need to perform colonoscopy to exclude tumors of colorectal origin and abdominocentesis to obtain mucinous ascites accompanied by elevated ascites CEA, which would most likely point to PMP of appendiceal origin. Moreover, incomplete cytoreduction, elevated ascites CEA, higher pathological grade and the more numbers of elevated ascites tumor markers predicted poor prognosis. Our findings contribute to the theoretical basis for establishing a clinical diagnosis and prognosis of PMP.

Importantly, the present study provides further evidence to support the importance of complete cytoreduction for patients with PMP. Unfortunately, the majority of PMP patients in China do not seek early medical advice and may be managed with an incorrect diagnosis. The tumor load of the study patients was relatively high, which resulted in a correspondingly high rate of CCR 2/3 resection. Chua et al. [17] conducted a large-scale retrospective multicenter study of 2298 patients that showed less than optimal debulking surgery (CCR 2 or 3; *P* < 0.001) was an independent predictor of poorer overall survival. Ansari et al. [18] retrospectively analyzed the clinical data of 1000 patients with PMP who underwent CRS combined with HIPEC. Not surprisingly, patients with complete cytoreduction (CCR-0 or CCR-1) had much higher 5- and 10-year survival rates than patients with CCR-2 or CCR-3 (87.4%, 70.3% vs. 39.2%, 8.1%, respectively). Prior studies also indicated that high-grade histology was another key prognostic factor associated with worse overall survival [19–21], which is in accordance with our results.

### Prognostic analysis of serum tumor markers in patients with PMP

A previous report identified that the size of lesions affected the sensitivity of CT examination, and the CT-PCI significantly underestimated the clinical PCI score [22]. The National Cancer Institute in Italy demonstrated that a normal preoperative level of CA125 was associated with adequate CRS and was a good prognostic factor for PMP patients. Moreover, elevated preoperative CA199 was an independent factor for predicting worse progression-free postoperative survival [23]. In a previous study, the result showed that only positive CA199 was an independent prognostic factor that affected the overall survival rate (*P* = 0.034). Furthermore, the 5- year survival rate of patients with CA199 > 1000 U/ml was 23%, whereas if the level was < 100 U/ml, the survival was dramatically higher at 90% (*P* < 0.001) [24]. Canbay studied 449 PMP patients and found that preoperative CEA level could predict disease progression, surgical treatment effect, progression-free survival, and overall survival [25]. Finally, elevated serum CEA, CA199 and CA125 was previously shown to reflect poor OS of appendiceal PMP [12]. In our study, serum CA199 and CA125 could predict overall survival for appendiceal PMP patients by univariate analysis. Taken together, previous literature reports have presented conflicting evidence regarding the prediction of prognosis for PMP patients by serum tumor markers. Therefore, it is essential to seek new markers.

### Diagnostic and prognostic value of markers in ascites for appendiceal PMP

It has been confirmed that PMP is rarely transmitted via the bloodstream. Peritoneal implantation and dissemination have been the main mechanisms of metastasis. Nummela found that CEA, the glycosylphosphatidylinositol-anchored glycoprotein, was probably shed from the surface of PMP cells into the mucinous ascites [26]. Recent study indicated that markers in serum and ascites could be used to judge the severity and predict the resectability for PMP [27]. However, whether markers in ascites could be used to identify the origin for PMP was not verified. In the present study, ascites tumor markers were more sensitive than serum tumor markers in the diagnosis of appendiceal PMP. CEA in ascites could provide adjunctive evidence for the origin of PMP and the more numbers of elevated ascites tumor markers can predict poor survival.

## Conclusion

Because PMP is relatively rare, and the imaging features are atypical, it is difficult to specify the exact cite of its origin preoperatively, especially in females. Therefore, it would be optimal to find a method that would help solve this dilemma. In the present study, CEA in ascites was found to be useful in identifying the origin of PMP. Furthermore, elevation of ascites CEA, high pathological grade and incomplete cytoreduction predicted poor prognosis of appendiceal PMP. These results should be valuable for clinicians managing this disease.

However, there were several limitations to the study. First, due to the limitations inherent in a retrospective study, some data were incomplete. Second, with the clinical application of ascites tumor markers, multicenter and larger scale studies will become possible.

In the future, focus on the molecular mechanism of ascites in PMP patients might provide therapeutic targets that would enhance patient prognosis. Also, we will focus on whether ascites tumor markers can be used as predictors of recurrence in patients after complete cytoreductive surgery.

## Data Availability

The datasets used and/or analyzed during the current study are available from the corresponding author on reasonable request.
